# Data on growth performance and survival of black scallops (*Mimachlamys varia* (Linnaeus, 1758)) reared under suspended conditions in waters of the Basque coast (SE Bay of Biscay)

**DOI:** 10.1016/j.dib.2022.108627

**Published:** 2022-09-20

**Authors:** Izaskun Zorita, José Germán Rodríguez, Joxe Mikel Garmendia

**Affiliations:** AZTI, Marine Research, Basque Research and Technology Alliance (BRTA), Herrera Kaia, Portualdea z/g, Pasaia 20110, Spain

**Keywords:** Pectinids, Scallop, Feasibility, Growing systems, Fattening phase, Offshore aquaculture, Longline, Cantabrian sea

## Abstract

This dataset presents the growth performance and survival of black scallops (*Mimachlamys varia*) reared under suspended conditions using different growing systems at two different sites of the Basque coast (SE Bay of Biscay). Monthly data on environmental variables (temperature, salinity, oxygen saturation and chlorophyll “a” concentration) in the water column is also provided. Juveniles obtained from a hatchery with a mean length of 23.6 ± 4.12 mm and a mean wet weight of 2.06 ± 1.09 g were deployed in cages and pots at two experimental sites: a raft installed in sheltered waters and a longline located at offshore waters. The experiment was run from June 2019 to August 2020 (447 days). Black scallops were collected every one or two months and at each sampling time a cage and a pot were retrieved from each site. Black scallop survival and growth performance were determined. This database is useful for (i) assessing the viability of the species on the Basque coast, (ii) determining the time needed for black scallops to reach commercial size, (iii) promoting species diversification in the emerging offshore aquaculture sector in the Basque Country and, (iv) for comparison with other biogeographical areas.

**Specifications Table**

**Table:** 

Subject	Agricultural Science, Aquaculture
Specific subject area	Black scallop growth and survival in suspended conditions
Type of data	Table
How the data were acquired	Biometric parameters (shell height and wet body and tissue weight) were measured in juvenile black scallops using a Vernier digital calliper to the nearest 0.1 mm and a digital balance (Fisher Scientific PPS2102) to the nearest 0.01 g as described in [1]. Furthermore, live and dead individuals cultured in each growing system (pots or cages) were counted to calculate survival rate. On the other hand, environmental variables (temperature, salinity, oxygen saturation and chlorophyll “a” concentration) were measured *in situ* at 3 m depth using a Sea-Bird CTD (Conductivity, Temperature and Depth) sensor.
Data format	Raw data
Description of data collection	Black scallop juveniles were deployed on a raft in sheltered waters and on a longline in offshore waters from June 2019 to August 2020. At each site, 160 individuals were introduced in each cage and 360 individuals in each pot. At each sampling carried out monthly or bimonthly, a cage and a pot were retrieved. Black scallop survival was calculated counting all live organisms and a subsample of 100 organisms was used for growth determination. Environmental variables were measured monthly at offshore waters.
Data source location	Region: SE Bay of Biscay. Basque coast.Latitude and longitude for collected samples/data of the two aquaculture experimental sites of the Basque coast: • Longline in offshore waters in Mendexa (43°21’23.40’’N, 2°26’54.00’’W) [2] • Raft in sheltered waters in the harbour of Mutriku (43°18’40.35’’N, 2°22’36.03’’W) [3]. Data: June 2019-August 2020.
Data accessibility [4]	Repository name: Zorita, Izaskun; Rodriguez, J German; Garmendia, Joxe Mikel (2022), “Database of growth and survival of black scallop (*Mimachlamys varia* (Linnaeus, 1758)) in the Basque coast”, Mendeley Data, V2. Data identification number: 10.17632/rptzp8pxx4.2. Direct URL to data: https://data.mendeley.com/datasets/rptzp8pxx4/1

**Value of the Data**

• The survival data presented herein are useful for understanding the feasibility of black scallop (*Mimachlamys varia*) in offshore waters of the Basque coast and is of importance for the diversification of mollusc species.
• Growth performance of black scallop presented in these data are useful for estimating the culture period necessary for commercial farming of black scallops on the Basque coast.
• Data on growth and survival of black scallops reared under different culture systems at two different sites are valuable for selecting the best location and culture system for black scallops on the Basque coast.
• Environmental variable data are helpful to understand the growth performance and survival of black scallops reared in offshore waters of the Basque coast.
• The dataset is useful for researchers in aquaculture and shellfish producers as information on black scallop culture under suspended conditions is scarce.

## 1 Data Description

Here we provide a dataset of the survival and growth performance of black scallops reared from June 2019 to August 2020 under different growing systems at two different sites of the Basque coast (SE Bay of Biscay), together with environmental variables data recorded in the water column. The raw data is available at https://data.mendeley.com/datasets/rptzp8pxx4/1.

The raw data consists of three tables:

Table “Growth”: Growth performance of black scallops reared under suspended conditions using different growing systems at two different sites of the Basque coast.

Table “Survival”: Survival of black scallops reared under suspended conditions using different growing systems at two different sites of the Basque coast.

Table “Environmental variables”: Environmental variables measured monthly in offshore waters of the Basque coast.

**Fig. 1:**
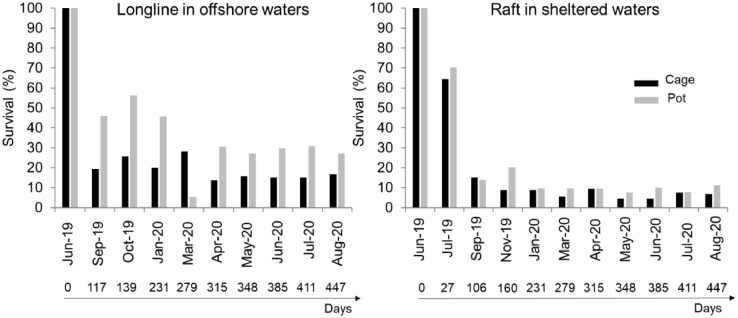
Survival (%) of black scallops reared on the longline in offshore waters and on the raft in sheltered waters from June 2019 to August 2020.

**Fig. 2:**
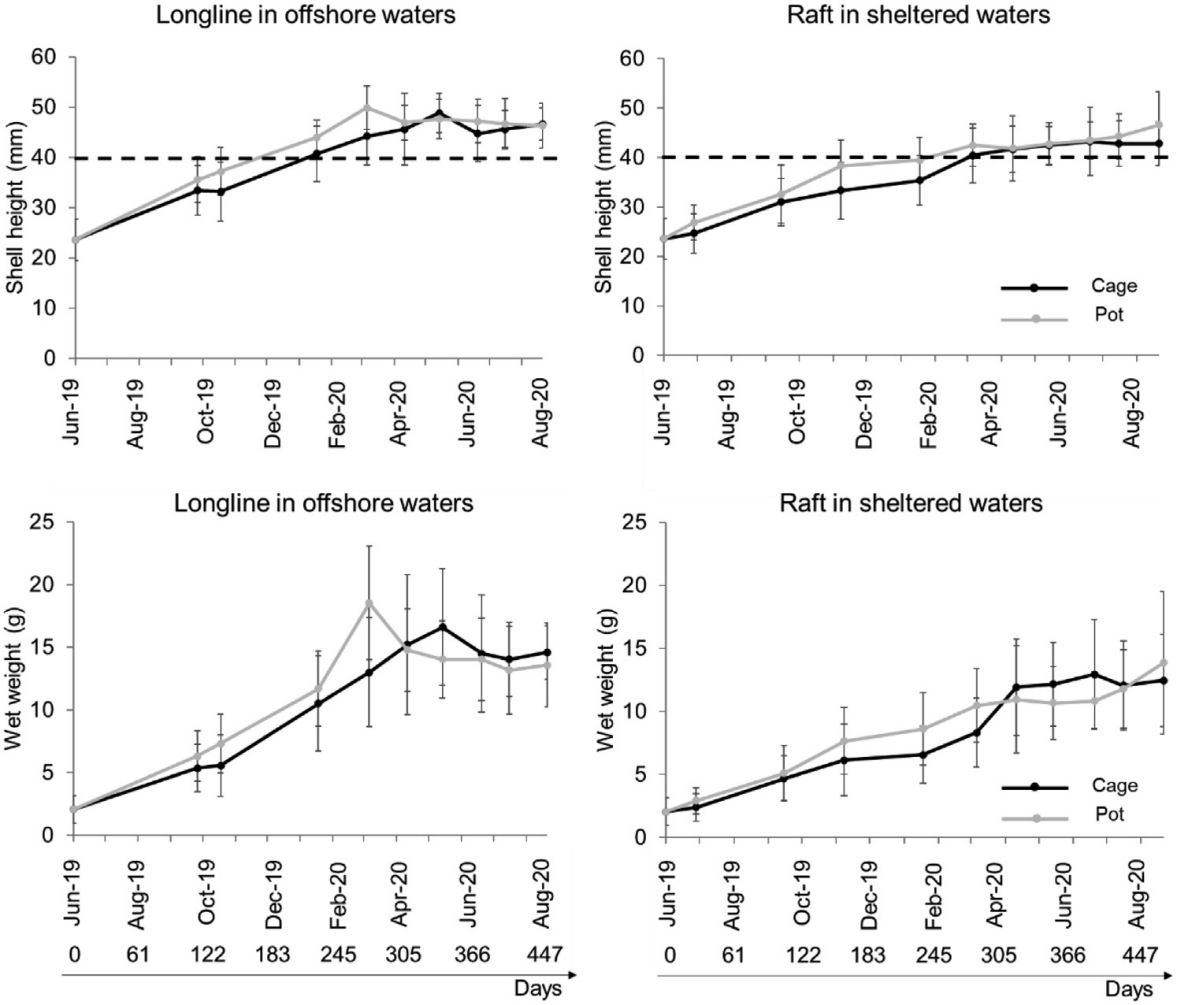
Evolution of mean ± standard deviation of shell height and wet weight of black scallops reared on the longline in offshore waters and on the raft in sheltered waters. The horizontal dotted line indicates commercial size.

The data allow to assess the survival (e.g., Fig. 1) and growth performance (e.g., Fig. 2) of black scallops reared under suspended conditions in waters of the Basque coast.

Black scallops reached commercial size in less than one year of culture on the Basque coast (starting from a mean length of 23.60 mm), which is consistent with another similar dataset of black scallops reared under suspended conditions in the Ionian Sea, Southern Italy [5].

## 2 Experimental Design, Materials and Methods

**Fig. 3:**
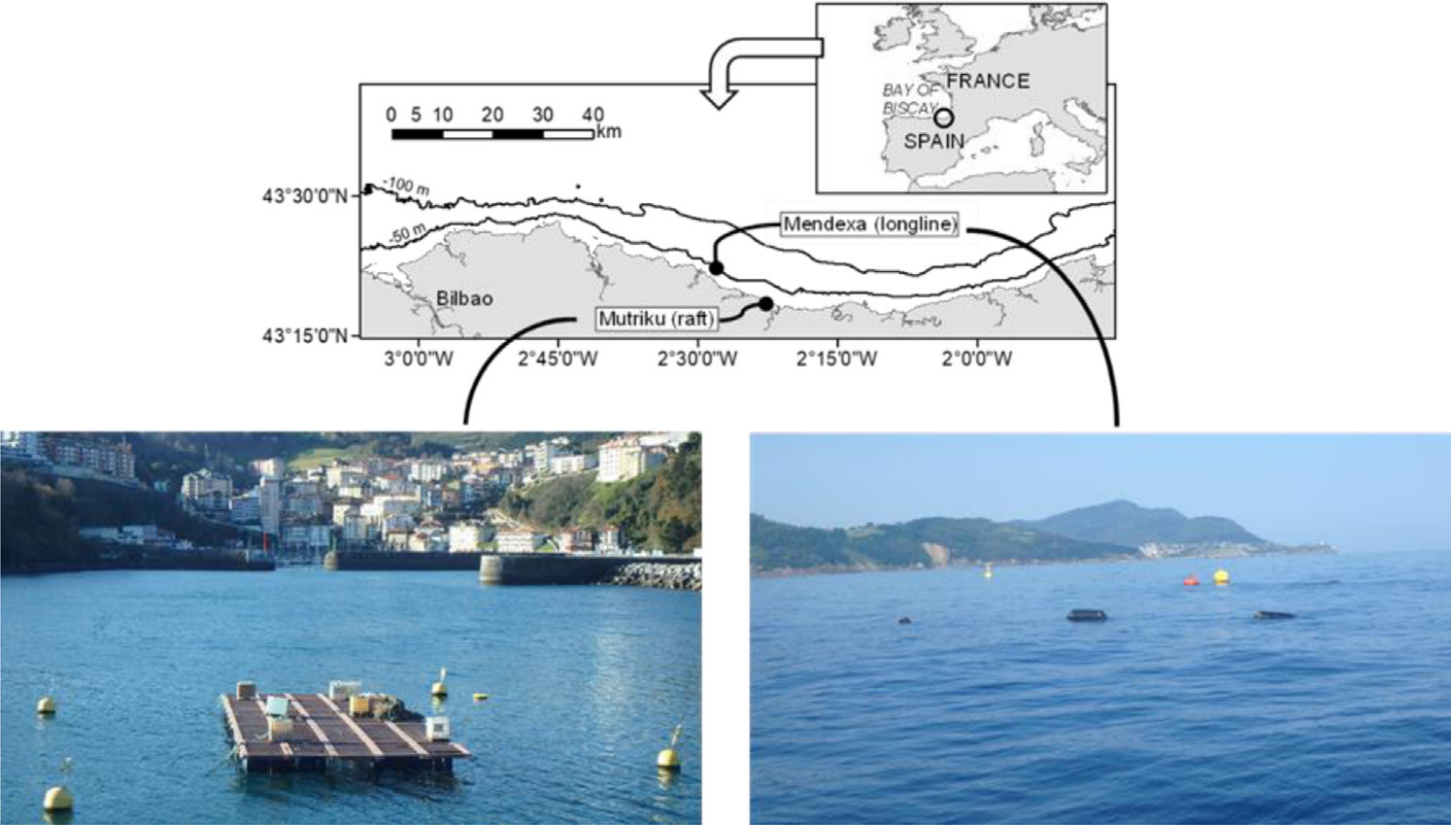
Location of the two experimental sites for aquaculture activities on the Basque coast (SE Bay of Biscay), a raft installed in sheltered waters in the harbour of Mutriku and a longline located in offshore waters of Mendexa, at two miles off the coast.

**Fig. 4:**
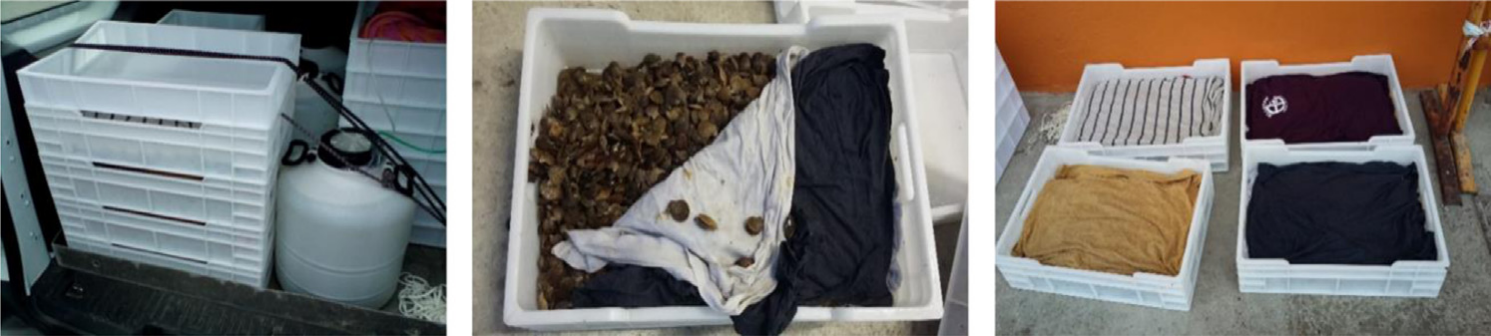
Transport of black scallops from Brittany to the Basque Country in boxes covered with towels moistened with seawater.

**Fig. 5:**
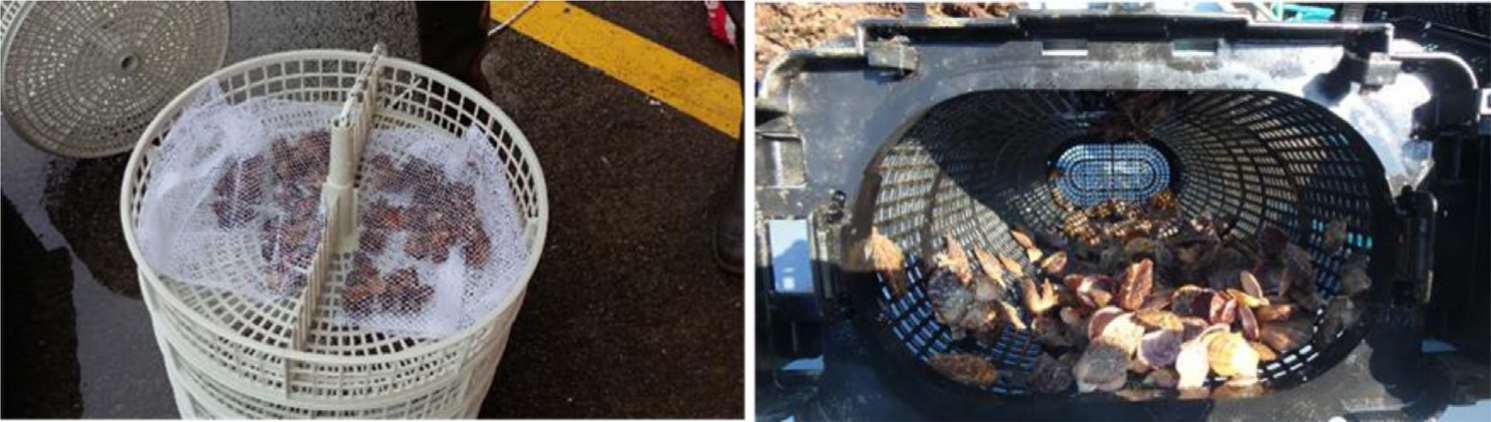
Distribution of black scallops in cages and in pots.

**Fig. 6:**
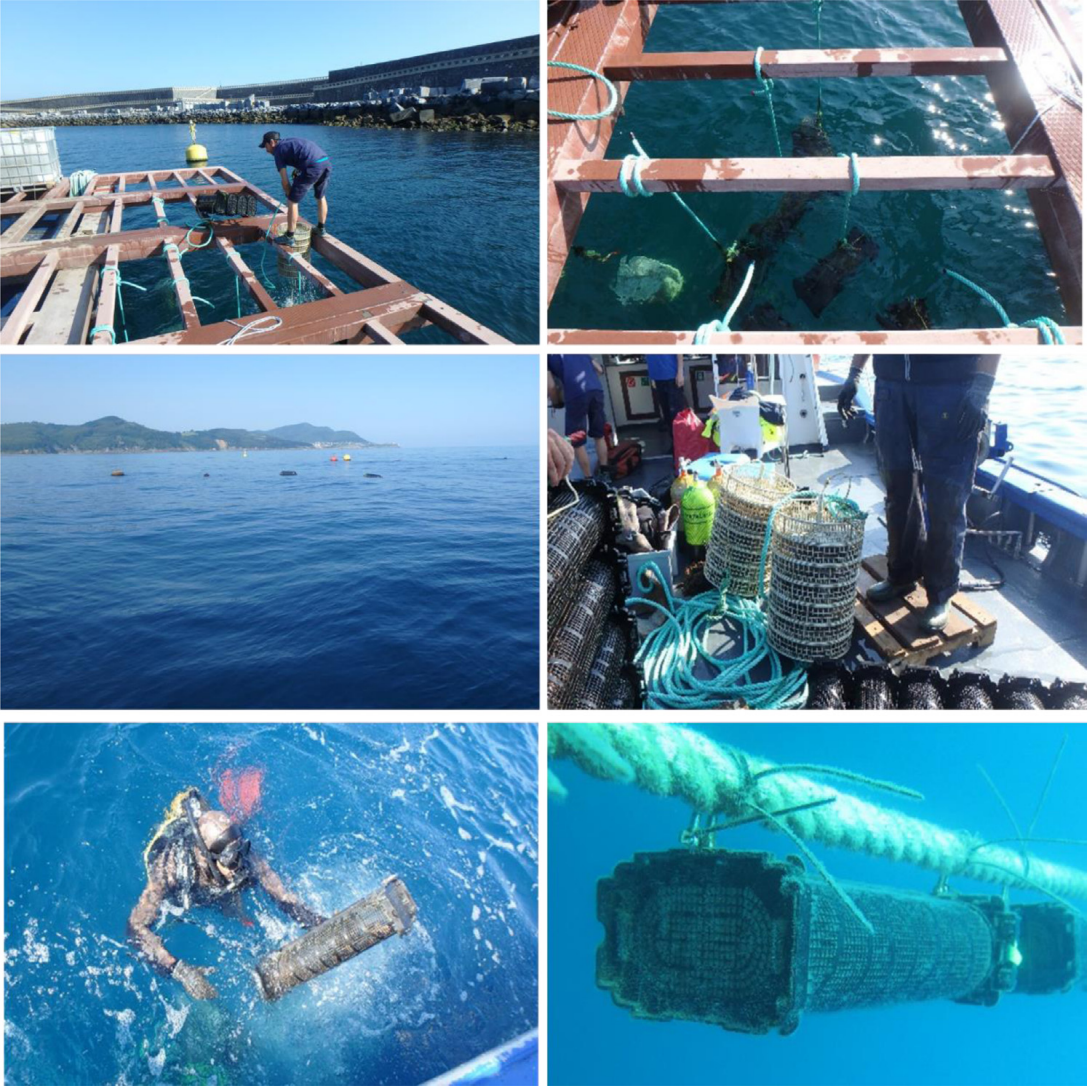
Installation of cages and pots containing black scallops at the two experimental sites.

**Fig. 7:**
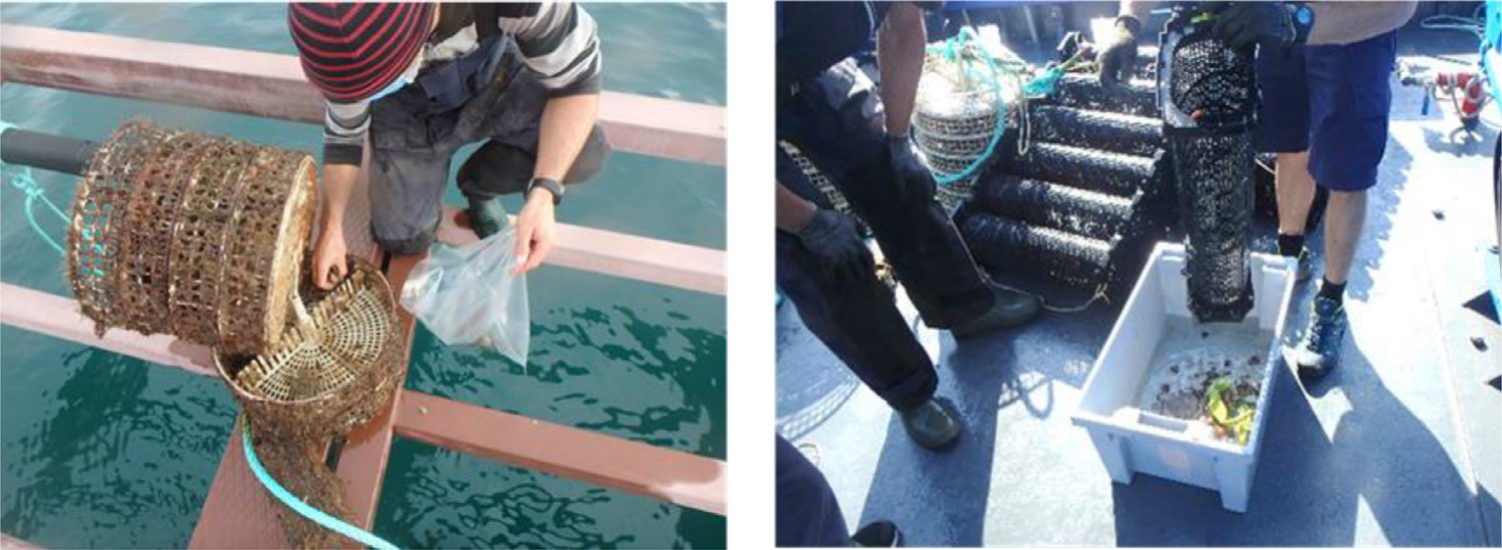
Retrieval of one cage and one pot from each site at each sampling time.

**Fig. 8:**
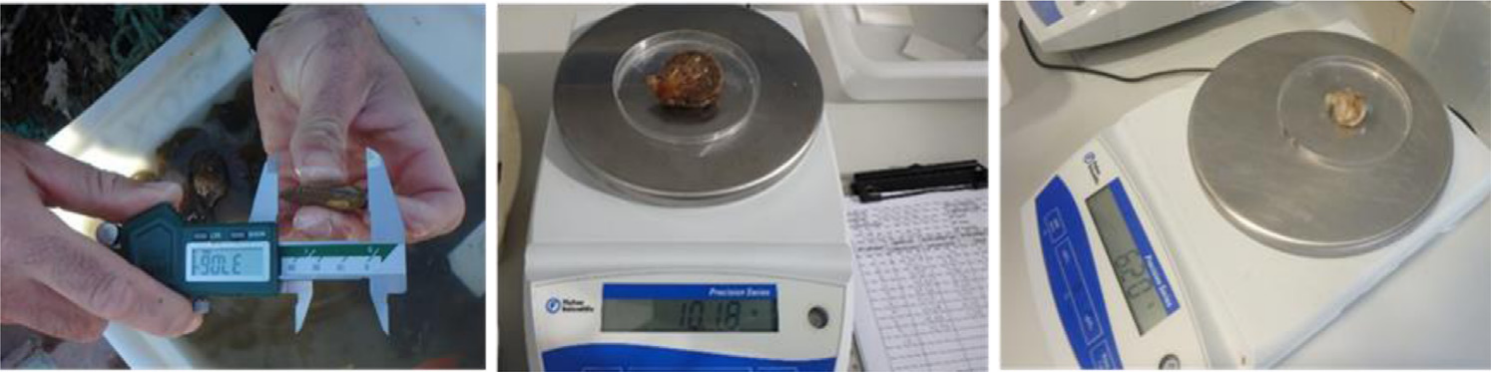
Determination of biometric parameters through the measurement of shell height and wet body and tissue weight.

The study was conducted at two experimental sites for aquaculture studies on the Basque coast: a raft installed in sheltered waters in the harbour of Mutriku, and a longline located in offshore waters of Mendexa, at two miles off the coast (Fig. 3). Black scallop juveniles were obtained from a hatchery in Brittany (France), with an initial mean length of 23.60 ± 4.12 mm and an initial mean wet weight of 2.06 ± 1.09 g. Black scallops were transported from Brittany to the Basque Country in boxes covered with towels moistened with seawater (Fig. 4). After a 12 h journey, black scallops were distributed in cages (415 mm diameter, 80 mm height, 8 mm mesh size) and pots (636 × 245 × 140 mm, 6 mm mesh size) (Fig. 5). 160 individuals were introduced in each cage, while 360 individuals were placed in pots, which is the equivalent to 1183 individuals m^−2^ in cages and 2246 individuals m^−2^ in pots, respectively. At each site, cages and pots with black scallops were deployed at 3 m depth (Fig. 6). The experiment was run from June 2019 to August 2020. Samplings were carried out approximately every one or two months, but the frequency was not exactly the same at both sites during the first months of the experiment. At each sampling time, one cage and one pot were retrieved from each site (Fig. 7) and survival was calculated counting live organisms with respect to the initial number of organisms. Subsequently, a subsample of 100 black scallops was used to determine the shell height using a digital calliper and the wet body and tissue weight with a balance (Fig. 8), except when live individuals were less than 100 (then all individuals were measured). On the other hand, environmental variables (temperature, salinity, oxygen saturation and chlorophyll “a”) were measured monthly on the longline at 3 m depth where black scallops were deployed using a CTD sensor.

## Ethics Statements

These data were collected complying ARRIVE guidelines.

## CRediT authorship contribution statement

**Izaskun Zorita:** Conceptualization, Funding acquisition, Investigation, Writing – original draft. **José Germán Rodríguez:** Conceptualization, Investigation, Formal analysis, Writing – review & editing. **Joxe Mikel Garmendia:** Conceptualization, Data curation, Visualization, Writing – review & editing.

## Declaration of Competing Interest

The authors declare that they have no known competing financial interests or personal relationships which have or could be perceived to have influenced the work reported in this paper.

## Data Availability

Growth performance and survival of black scallops together with environmental variables (Original data) (Mendeley Data).

## References

[1] P. Iglesias, Estudio de la reproducción y reclutamiento de los pectínidos de interés comercial de las rías gallegas. PhD Thesis, Universidad de Santiago de Compostela (2012) pp. 337.

[2] Azpeitia K., Ferrer L., Revilla M., Pagaldai J., Mendiola D. (2016). Growth, biochemical profile, and fatty acid composition of mussel (*Mytilus galloprovincialis* Lmk.) cultured in the open ocean of the Bay of Biscay (northern Spain). Aquaculture.

[3] Bilbao J., Muñiz O., Rodríguez J.G., Revilla M., Laza-Martínez A., Seoane S. (2021). Assessment of a sheltered euhaline area of the southeastern Bay of Biscay to sustain bivalve production in terms of phytoplankton community composition. Oceanologia.

[4] Zorita I., Rodriguez J.G., Garmendia J.M. (2022). Database of growth and survival of black scallop (*Mimachlamys varia* (Linnaeus, 1758)) in the Basque coast. Mendeley Data.

[5] Prato E., Biandolino F., Parlapiano I., Papa L., Denti G., Fanelli G. (2020). Estimation of growth parameters of the Black Scallop *Mimachlamys varia* in the Gulf of Taranto (Ionian Sea, Southern Italy). Water.

